# Potential role of broad-spectrum azoles as therapy for *Malasezzia* bloodstream infection

**DOI:** 10.1016/j.mmcr.2023.05.002

**Published:** 2023-05-12

**Authors:** Sunish Shah, M. Hong Nguyen

**Affiliations:** a Antibiotic Management Program, University of Pittsburgh Medical Center, Pittsburgh, PA, United States; b Department of Pharmacy, University of Pittsburgh Medical Center, Pittsburgh, PA, United States; c Department of Medicine, University of Pittsburgh, Pittsburgh, PA, United States

**Keywords:** *Malassezia*, Azoles, Posaconazole, Amphotericin, Fungemia

## Abstract

Amphotericin B is the currently recommended therapy for *Malassezia* invasive infection (MII), but this drug requires intravenous administration and is associated with significant toxicity. The role of broad-spectrum azoles in managing MII is not clear. We describe two cases of MII due to *M. pachydermatis* and *M. furfur* that were successfully treated with posaconazole and reviewed the literature to assess the position of posaconazole in treating MII.

## 1 Introduction

*Malassezia* species are commonly associated with benign skin conditions such as tinea versicolor and folliculitis. However, among immunocompromised patients or those requiring total parenteral nutrition, this lipophilic yeast can cause invasive disease. Amphotericin B (amB) products are the current standard therapeutic approach to *Malassezia* invasive infection (MII) [1]. Broad-spectrum triazoles, however, are not recommended for MII by current consensus guidelines [1]. We reported two patients with MIIs that were successfully treated with posaconazole and performed a background literature review to assess the role of broad-spectrum azole in treating MII.

## 2 Case presentations

**Case 1**: A 60-year-old man with a history of ruptured abdominal aortic aneurysm which was complicated by aortoenteric fistula. He underwent partial resection of the duodenum and jejunum for small bowel ischemia, repair of aortoenteric fistula and evacuation of intra-abdominal hematoma five months prior to admission. The patient was placed on chronic total parenteral nutrition (TPN) post-operatively. He was electively admitted for an attempt to restore intestinal continuity. As part of pre-operative evaluation, a set of blood cultures was drawn which grew a yeast that was identified as *M. pachydermatis* by matrix-assisted laser desorption/ionization time-of-flight mass spectrometry (MALDI-TOF MS) (BioMérieux). Three sets of blood cultures obtained daily until day 3 (2 from peripheral blood draw, and 1 from central line) all grew *M. pachydermatis*. The central line was removed, TPN was stopped, and the patient was started on posaconazole 300 mg intravenously (IV) daily. Subsequent peripheral blood cultures were sterile. The minimal inhibitory concentrations (MICs) of amB, caspofungin and fluconazole were 0.06 μg/mL, 4 μg/mL, 4 μg/mL, respectively. The MICs of posaconazole, isavuconazole and voriconazole were all <0.03 μg/mL. A transthoracic echo demonstrated lesions suggestive of Lambl's excrescenses on the patient's mitral valve chordae and automatic implantable cardioverter-defibrillator (AICD). The patient was treated with 6 weeks of posaconazole. Serial posaconazole troughs obtained during therapy ranged from 1.3 to 2.7 μg/mL. The patient was alive with no recurrence of *Malassezia* infection at 3 years of follow-up.

**Case 2**: An 80 year-old woman with short gut syndrome requiring chronic TPN presented to an outside hospital with abdominal pain and fatigue. Within the previous 9 months, the patient had received several courses of caspofungin for candidemia and intraabdominal candidiasis. Blood cultures identified yeast on Gram stain, which did not grow on Sabouraud dextrose agar. *M. furfur* was subsequently grown on Sabouraud's dextrose agar plate supplemented with cycloheximide and overlaid with olive oil. The patient was subsequently transferred to our institution for further management. Upon transfer, the patient was hemodynamically stable, afebrile, and without leukocytosis. Follow-up blood cultures were sterile following central line removal and treatment with 3 mg/kg of IV liposomal amB (L-amB). On day 3, the patient developed acute kidney injury, and she was transitioned to IV posaconazole 300 mg every 24 h to complete a 14-day course. The patient was deemed clinically cured prior to discharge. At 1 month of follow-up, she had no recurrence of *Malassezia* infection. Long-term follow-up was not available.

## 3 Discussion

MIIs have increasingly been reported, especially among premature neonates and immunocompromised hosts. To date, the optimal therapy for MII is unclear because of the infrequency of the disease and the absence of interpretive guidelines for in-vitro activity. The current treatment recommendations, therefore, are based on published anecdotal experience through which amB in conjunction with vascular catheter removal was utilized [[2], [3], [4], [5]]. Unfortunately, many of the MII cases were reported in the era before systemic azole antifungals were commercially available. Nevertheless, for the management of MII, the European Confederation for Medical Mycology recommends L-amb as a first line agent, amB as a second line agent, and does not recommend treatment with an azole antifungal [1].

We performed a literature review of articles written in English using MEDLINE, PUBMED, and EMBASE, using the search terms “*Malassezia* fungemia”, “*Malassezia* bloodstream infections”, and “systemic *Malassezia* infections” between January of 1985 and January 2021. We identified 53 patients with *Malassezia* in blood cultures. Of these 53 patients, 13 patients received an azole antifungal therapy for treatment. Among these 13 patients, 8 received azole monotherapy, 4 received sequential therapy with a short duration of amB followed by an azole, and 1 patient combined amB and fluconazole (Table 1). In this review, we defined uncomplicated fungemia if there was 1) no evidence of endovascular infection, 2) no implanted devices or prostheses, 3) no evidence of deep-seated infection, 4) source control, and 5) clinical and microbiologic clearance after 5 days of therapy. Of the 8 patients treated with mono-azole therapy, 5 received miconazole, 2 received fluconazole and our patient was treated with posaconazole [2,3]. Of note, none of these 8 patients received immunosuppressive therapy. With the exception of our patient, all of the previously reported cases who were managed with mono-azole therapy were neonates. In addition these patients had TPN-associated *Malassezia* fungemia. All except one had uncomplicated fungemia; our patient had complicated fungemia because of retained AICD. Among the 4 patients receiving sequential therapy, 2 had uncomplicated (one with TPN-associated infection and one with likely central-line associated fungemia) and 2 had complicated fungemia (one had endocarditis and one had septic arthritis) [[6], [7], [8]]. The 2 patients with uncomplicated fungemia received amB for 3 and 10 days, followed by 11 days of posaconazole and 4 days of fluconazole, respectively [6]. The remaining 2 patients received at least 2.5 weeks of amB, followed by either ketoconazole or voriconazole [7,8]. The single patient receiving combination of both amB and fluconazole had complicated fungemia with osteomyelitis [4]. Altogether, only 1 patient died, and the remaining patients had successful therapy and survived.

**Table 1:** Patients treated with exclusive, sequential, or combination Azole antifungals for invasive *Malassezia*.

Reference	Prior Azole exposure	Age/Sex	Species	Source of *Malassezia* fungemia	Treatment	Microbiologic cure	Mortality described in report
Exclusive azole treatment							
Case 1	14 days of caspofungin/fluconazole 8 months prior	60/M	*M. pachydermatis*	TPN^b^ (line removed)	Posaconazole X 4 weeks	Yes	No
Surmont et al., 1989 [2]	No	Infant/M	*M. furfur*	TPN (line removed)	IV miconazole X 10 days	Yes	No
	No	Infant/M	*M. furfur*	TPN (line removed)	IV miconazole X 15 days	Yes	No
	No	Infant/M	*M. furfur*	TPN (line removed)	IV miconazole X 14 days	Yes	Yes
	No	Infant/M	*M. furfur*	TPN (line removed)	IV miconazole X 14 days	Yes	No
	No	Infant/F	*M. furfur*	TPN (line removed)	IV miconazole X 14 days	Yes	No
Chryssanthou et al., 2001 [3]	No	Infant/NR	*M. pachydermatis*	TPN (line probably removed)	Fluconazole (duration NR)	Yes	No
	No	Infant/NR	*M. pachydermatis*	TPN (line probably removed)	Fluconazole (duration NR)	Yes	No
**Sequential amB then azole therapy**							
Chu et al., 2002 [6]	None	39/M	*M. furfur*	Unknown	AmB^a^ X 10 days; Sequential fluconazole X 14 days due to amB hypokalemia	Yes	No
Wurtz et al., 1988 [8]	None	70/M	*M. furfur*	Septic Hip	AmB X 2.5 weeks; Sequential ketoconazole X 3.5 weeks	Yes	No
Granok 2020 [7]	None	33/M	*M. furfur*	IVDA^^^/endocarditis	Preoperative: L-amB + Voriconazole Post-operative: Sequential voriconazole >60 days due to L-amB nephrotoxicity	Yes	No
Case 2	Several courses of caspofungin 9 months prior	80/F	*M. furfur*	TPN (line removed)	L-amb X 3 days; Sequential posaconazole X 11 days	Yes	No
**Combination treatment**							
Pedrosa et al. [4]	No	2/F	*M. furfur*	Central line (removed)/osteomyelitis	Fluconazole + L-amb synergy X 50 days	No- subsequent development of pityriasis versicolor	No

a Amphotericin B; ^^^Intravenous Drug Abuse.

b Total parenteral nutrition.

It is important to note that breakthrough *M. pachydermatis* fungemia has been reported in a leukemic patient receiving posaconazole for prophylaxis in the setting of neutropenia [9]. While it is not unusual for patients experiencing prolonged neutropenia to suffer from breakthrough invasive fungal infections that display in-vitro activity to the azole used for prophylaxis, the optimal therapy for disseminated *Malassezia* infections remains even less clear in this patient population [10]. Nevertheless, it is worthwhile to note that a tentative azole epidemiological cut-off values (ECVs) have been determined for *M. furfur* and *M. pachydermatis* [11]. Based on ECVs, over 90% of *Malassezia* isolates were within the susceptible population for all azoles except for fluconazole to which the MIC is somewhat higher. Echinocandins appear to have no activity against *Malassezia* spp.

Altogether, our review suggests that in a subset of patients with *Malassezia* fungemia, such as those with central line and/or TPN-associated infection or those with uncomplicated fungemia, central line removal and a mold-active azole such as posaconazole, voriconazole or itraconazole could provide a more tolerable therapeutic option. Given a more favorable tolerability and safety profile, and generally lower MICs, posaconazole may be preferred over voriconazole.^12^

## Conflicts of interest

There are none.
